# Auxiliary rapid identification of pathogenic and antagonistic microorganisms associated with *Coptis chinensis* root rot by high-throughput sequencing

**DOI:** 10.1038/s41598-021-90489-9

**Published:** 2021-05-27

**Authors:** Hailang Liao, Ling Huang, Na Li, Wenjia Ke, Yiqing Xiang, Yuntong Ma

**Affiliations:** 1 State Key Laboratory Breeding Base of Systematic Research, Development and Utilization of Chinese Medicine Resources, Chengdu University of Traditional Chinese Medicine, Chengdu, 611137 China; 2 Department of Pharmacy, Chengdu University of Traditional Chinese Medicine, Chengdu, 611137 China; 3 The Engineering and Technology Research Center for the Protection and Development of Yalian Resourcesin Sichuan Province, Meishan, 620360 Sichuan China

**Keywords:** Microbiology, Plant sciences, Pathogenesis

## Abstract

Root rot reduces the yield and medical quality of *C. chinensis* (Cc). Previous studies of *Coptis* root rot focused on the identification of pathogens and the rhizosphere microbial community composition. The present study aimed to identify potential pathogenic and antagonistic microorganisms associated with root rot based on a high-throughput sequencing technique to prevent this disease. Healthy and diseased Cc in the endosphere and rhizosphere from the same field were collected to investigate the differences in microbiome composition and function. The results showed that the composition and function of microbes were different. The numbers of animal pathogens, soil saprotrophs, plant saprotrophs, and wood saprotrophs in the endosphere of diseased Cc were higher than those in the healthy endosphere and were dominated by Phaeosphaeriaceae, *Cladorrhinum*, *Fusarium*, *Exophiala*, and Melanommataceae. *Fusarium*, *Volutella*, *Cladorrhinum*, *Cylindrocarpon*, and *Exophiala* were significantly enriched in the endosphere of the diseased plants. Co-occurrence network analysis showed that *Bacillus* was negatively correlated with *Fusarium*, *Volutella*, and *Cylindrocarpon*, indicating that *Bacillus* may be antagonistic microorganisms. To verify the sequencing results, *F. solani* and *F. avenaceum* were isolated and verified as pathogens, and 14 *Bacillus* strains were isolated, which displayed an apparent suppression effect against the two pathogens on PDA medium and detached roots. The strategy of high-throughput sequencing has the potential for the comprehensive identification of pathogenic and antagonistic microorganisms for plant disease. These results provide research ideas and microbial resources for future studies on mitigating or preventing root rot damage to Cc.

## Introduction

Coptidis rhizoma (HuangLian) is a commonly used medicinal material in China, that is derived from dry rhizomes of *Coptis chinensis* Franch, *C. deltoidea* C. Y. Cheng et Hsiao or *C. teeta* Wall. Due to the excessive mining of medicinal materials, the wild resources of Coptidis rhizoma have been exhausted. The demand for *Coptis* medicinal materials has risen sharply. As a result, the cultivation of Cc has been the main source of these medicinal materials. However, Cc has been planted in large areas with high density in different regions, and the abuse of chemical fertilizers and pesticides has led to the frequent occurrence of diseases in cultivated *Coptis* plants, mainly manifested by the high incidence of soil-borne disease root rot.

According to previous surveys^1, 2^, the annual incidence of *Coptis* root rot in Shizhu, the largest production area of Cc in China, is 10–20%, and the incidence in areas with severe disease is 60–90%, with no harvest. When root rot of *Coptis* plants occurs, the leaves of infected plants show wilt, necrotic lesions, drying, and death. The fibrous roots and rhizomes exhibit brown discoloration and progressive necrosis that caused mortality of the infected plants. *Fusarium solani* was first reported as the pathogen causing Cc root rot^3^, which was confirmed by Chen Shanshan et al.^4^. Recently, new *Fusarium* fungi have been reported as pathogens causing *Coptis* root rot, mainly including *F. carminascens*^5^, *F. oxysporum*^6^, *F. tricinctum*^6^, and *F. avenaceum*^7^. However, many obligate intracellular pathogens do not grow in pure culture and never form reproductive structures, which renders their detection and identification difficult. Rapid and accurate identification of pathogenic microorganisms is essential for the detection and employment of appropriate mitigation measures.

To date, there are no effective prevention and control measures for *Coptis* root rot. Farmers generally adopt measures such as pulling out diseased plants, replacing soil, and irrigating potassium permanganate, but effects of these measures are not obvious^2^. Research and development of new *Coptis* root rot prevention methods are imminent. In recent years, the use of antagonistic microorganisms as biocontrol agents have attracted special attention because they are a method of plant disease management that has a minimal impact on the environment^8–12^. In nature, there are various microorganisms in the growth environment of plants that attach to the surface and inside the plants. These microorganism groups are collectively referred to as the plant microbiome, which can promote plant growth, inhibit disease occurrence, regulate plant microecological structure^13, 14^, and become an important determinant of plant health and growth^15, 16^. The interspecies interaction of the plant microbiome increases the host's barrier against external pathogen colonization, thereby playing an important role in protecting the host's health^17, 18^. Inoculation with native bacteria can significantly reduce the incidence of field diseases without affecting plant growth^19^. Root-related bacteria of healthy plants can affect the abundance and diversity of root filamentous eukaryotic microorganisms, thereby protecting the host from diseases caused by fungi and oomycetes^20^.

High-throughput sequencing technology is a powerful tool to reveal the flora of plant diseases. This technique can detect plant pathogenic microorganisms and discover beneficial microorganisms that may inhibit plant pathogens and promote host growth, therefore providing possible solutions for plant pathogen infection and prevention^21–26^. Liu et al.^21^ used 16S rRNA and ITS sequencing to identify the endophytic and rhizosphere microbes involved in ginseng rust roots. Ou et al.^24^ used Illumina metabarcoding to trace changes in microbiome composition upon pathogen invasion over time, to investigate the expected changes in the microbiome between conductive and suppressive soil upon pathogen invasion and to identify potential microbial agents that induce soil suppression against *Fusarium* wilt disease.

Compared with previous studies, not only the rhizosphere but also the endosphere microbial differences between healthy and diseased plants were compared by amplicon sequencing. More importantly, we compared the differences between microbial composition and function, looked for indicator microorganisms, and constructed a microbial network to infer the interactions between microorganisms. To verify the reliability of the evaluation method, the pathogen and beneficial microorganisms were isolated, identified, and functionally verified. This study not only helps clarify the fundamental cause of root rot but also benefits the discovery of antagonistic microorganisms for *Coptis* root rot prevention or control.

## Results

### Sequencing data and OTU clustering

**Figure 1:**
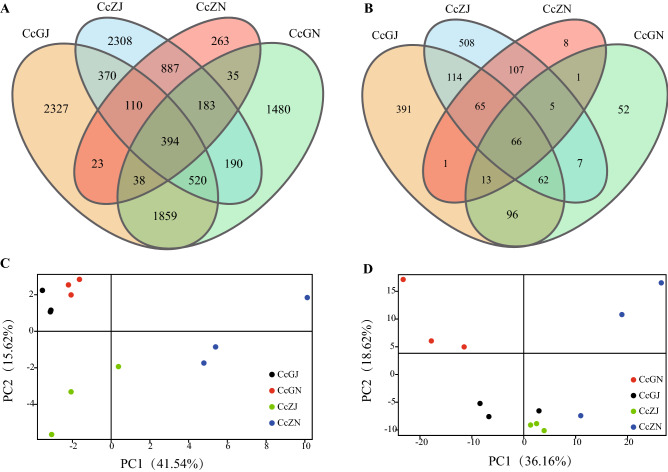
OTU variance analysis of microbes in the rhizosphere and endosphere of healthy and diseased Cc*.* (**A**) Venn diagram of bacteria; (**B**) Venn diagram of fungi; (**C**) PCA of the bacterial community; (**D**) PCA of the fungal community.

After using high-throughput sequencing technology to remove low-quality, barcode and primer sequences, we finally obtained 1,369,013 valid 16S sequences from 12 samples for subsequent analysis. The sequence lengths of valid sequences were distributed between 302 and 476 nt, where N50 > 443 nt and N90 > 441 nt. Then, the host chloroplast gene, mitochondrial gene, and the total number of tags that did not exceed 5 sequences were removed. The four groups produced a total of 10,989 OTUs, of which the CcGJ group (the rhizosphere of diseased Cc), CcZJ group (the rhizosphere of healthy Cc), CcGN group (the endosphere of diseased Cc), and CcZN group (the endosphere of healthy Cc) contained 3805, 3060, 3315, and 1768 OTUs, respectively. The numbers of OTUs unique to the CcGJ group, CcZJ group, CcGN group, and CcZN group were 1585, 1239, 1123, and 369, respectively. Principal component analysis (PCA) showed that the CcGJ group and the CcGN group were relatively similar, and the differences between the other groups were larger. This means that the rhizosphere of the diseased Cc had a similar bacterial composition. The detailed results are shown in Tables S1–S3 and Fig. 1A,C.

There were 2,041,477 valid ITS sequences used for subsequent analysis. The valid sequence lengths ranged from 204 to 399 nt, among which N50 > 340 nt and N90 > 327 nt. The host ITS2 gene and the total number of tags that did not exceed 5 sequences were removed, and 1,496 OTUs were generated. Among them, the CcGJ group, CcZJ group, CcGN group and CcZN group contained 808, 934, 302, and 266 OTUs, respectively. The numbers of OTUs unique to the CcGJ group, CcZJ group, CcGN group and CcZN group were 391, 508, 52, and 8, respectively. PCA found that the CcGJ group and the CcZJ group were similar, and differences between other groups were greater, indicating that the rhizosphere of the diseased and the healthy plants had a similar fungal composition. The detailed results are shown in Tables S4-S6 and Fig. 1B,D.

### Composition of and environmental influence on the coptis root microbial community

The Shannon index (Figure S1) showed that there were significant differences in the richness and uniformity of bacterial OTU levels in the endosphere of diseased and healthy plants, and there were significant differences in the richness and uniformity of OTU levels of rhizosphere fungi between diseased and healthy plants.

β-Diversity analysis showed that the root microbiome of Cc was affected by disease and niche. In this study, the Bray–Curtis distance among samples was analyzed by principal coordinate analysis (PcoA) and nonmetric multidimensional scaling (NMDS) (Figure S2). The results are displayed on the first principal axis (Pco1/MDS1), and the root microbiome of Cc was divided into two clusters according to whether the plant was diseased or healthy, indicating that the disease will cause drastic changes in the root microbiome of Cc. On the second axis, the microbiome was divided into two clusters within the rhizosphere, which indicated that different ecological niches had a great influence on the root microorganisms of Cc.

### Fungal and bacterial taxa in healthy and diseased coptis roots

After species annotation, a total of 40 bacterial phyla were identified. The top 10 most abundant species are shown in Figure S3. From the figure, the community composition and structure of the four groups are significantly different. Compared with healthy plants (CcZJ and CcZN groups), the relative abundance of Gemmatimonadetes and Parcubacteria in the endosphere of diseased plants (CcGN groups) increased significantly, Bacteroidetes increased significantly in the rhizosphere of diseased plants (CcGJ group), and the relative abundance of Acidobacteria and Chlamydiae decreased significantly in the endosphere and rhizosphere of the diseased plants (CcGN and CcGJ groups).

A total of 6 fungal phyla were identified (Figure S4). The relative abundance of Ascomycota fungi in the rhizosphere of healthy and diseased plants was the highest (over 75%). Compared with healthy plants (CcZJ and CcZN groups), Chytridiomycota in the endosphere were significantly reduced in the diseased plants (CcGN group). In the rhizosphere, Rozellomycota and Chytridiomycota were significantly reduced in the diseased plants (CcGJ group) compared to the healthy plants.

### Functional analysis of microbes in healthy and diseased coptis roots

**Figure 2:**
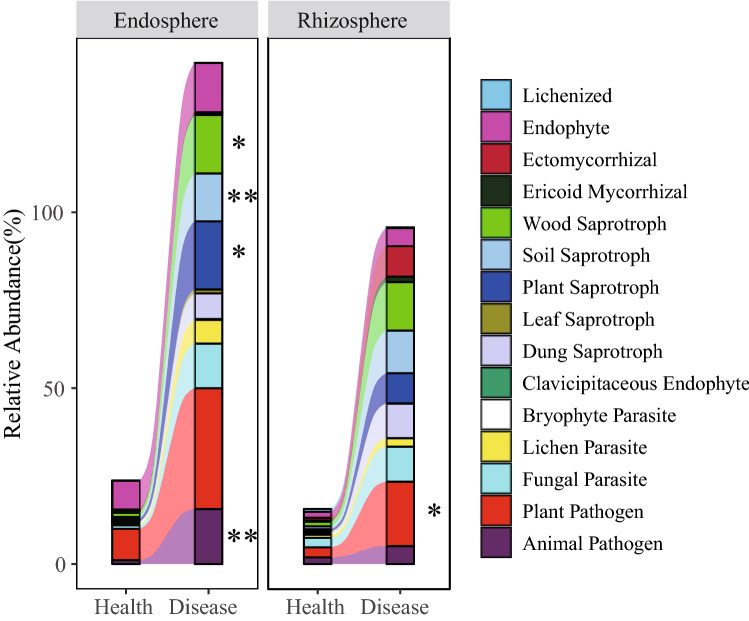
Stacked shock map of the functional group distribution of the fungal community in the rhizosphere and the endosphere of diseased and healthy Cc*.* *0.01 < *P* ≤ 0.05; **P ≤ 0.01 (Welch's t test).

Funguild is an efficient method for analyzing fungal community data sets^27^. It was used to predict the nutrient and functional groups of fungal communities in the rhizosphere and endosphere of diseased and healthy Cc. The results showed that the fungal community was divided into three nutrition pattern groups (the sequences identified as multiple nutritional patterns were repeated calculations), among which the saprotroph of the diseased endosphere was significantly higher than that of the healthy endosphere (*P* = 0.01439). These fungi might be an important factor leading to the pathogenesis of Cc root rot. Further analysis of the functional groups of the fungal community in the endosphere of Cc (Fig. 2) showed that the animal pathogens and the soil saprotroph in the endosphere of the diseased Cc were significantly higher (*P* ≤ 0.01) than those in the healthy endosphere. Plant saprotrophs and wood saprotrophs were significantly (0.01 < *P* ≤ 0.05) higher than those in the roots of healthy endospheres. The plant pathogens in the rhizosphere of diseased Cc were significantly higher (0.01 < *P* ≤ 0.05) than those in the rhizosphere of healthy Cc. The top 5 fungal types with significant differences in relative abundance in the endosphere of diseased plants compared to healthy plants included Phaeosphaeriaceae, *Cladorrhinum*, *Fusarium*, *Exophiala*, and Melanommataceae. The top 5 fungal groups with significant differences in the rhizosphere of diseased plants compared to the healthy plants were Phaeosphaeriaceae, *Clonostachys*, *Fusarium*, *Phialophora*, and Chaetothyriaceae.

**Figure 3:**
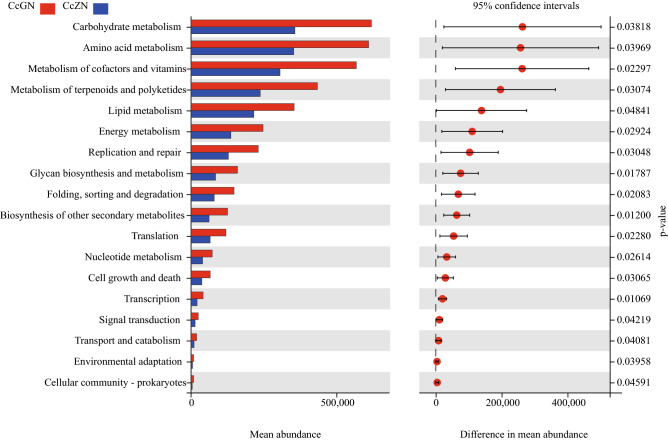
Prediction and analysis of significant differences between bacterial community distributions in the endospheres of healthy and diseased Cc*.* The pathways were predicted based on 16S sequences. The predicted metabolic pathways belonging to ‘Organismal Systems’ and ‘Human Diseases’ in the KEGG database were discarded.

Based on 16S rRNA gene information and PICRUSt2 analysis^28^, we predicted the functional potential of the subgenome of bacterial communities. The results showed that the related prediction pathways belonging to the "tissue system" and "human diseases" had been removed, and there was no significant difference in the function of the bacterial community predicted in the rhizosphere of healthy and diseased Cc; however, there were significant differences in the predicted bacterial community function within the endosphere (Fig. 3). A total of 18 pathways (KEGG level 2) were predicted in the roots of diseased *Coptis* plants, which were significantly higher than those of healthy plants.

### Indicator species analysis

**Figure 4:**
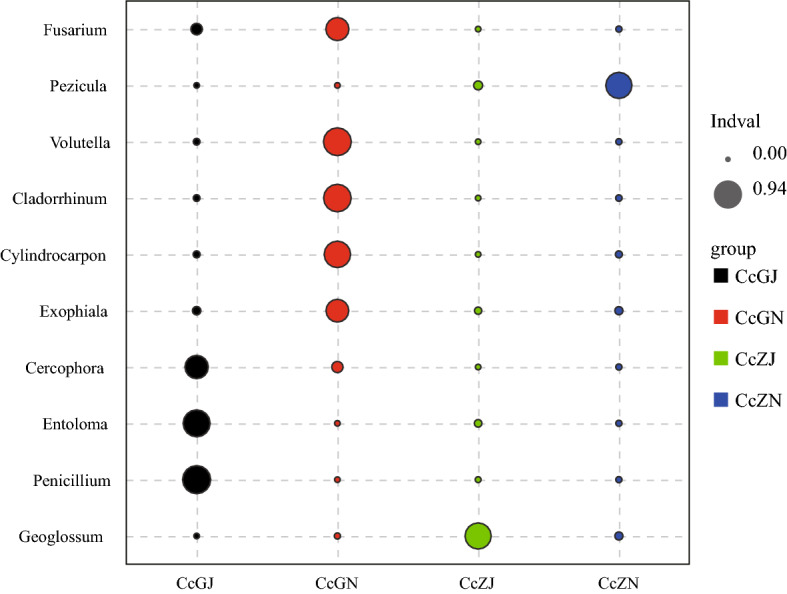
Analysis of indicator species of fungi in the rhizosphere and endosphere of healthy and diseased Cc*.* Node size represents the average relative abundance of one genus in each sample type.

The R project “labdsv” package was used to calculate the indicator value of each group for species with an abundance value > 0 and a total proportion > 0.1% in each sample of the comparison groups. Cross-validation of the statistical test was used to obtain the *P* value. The data are displayed as a bubble chart, and the biomarker of each group can be found intuitively by the size of the bubble. *Fusarium*, *Volutella*, *Cladorrhinum*, *Cylindrocarpon*, and *Exophiala* were significantly enriched in the endosphere of diseased Cc (Fig. 4). Accounting for 3.34–12.58%, 2.08–12.89%, 0.92–11.79%, 0.40–7.08%, and 0.72–2.74% of the total fungal OTUs for each sample. As shown in Figure S5, many bacteria were significantly enriched in the endosphere of the diseased Cc, such as *Flavobacterium*, *Sphingobium*, *Chryseobacterium*, and *Brevundimonas*, accounting for 1.75–5.38%, 0.30–0.54%, 0.02–2.08%, and 0.28–2.93% of the total bacterial OTUs for each sample. *Bacillus, Collimonas, Rhizobium, Aquincola, Acidicapsa, and Edaphobacter* were enriched in the endosphere of healthy Cc*,* accounting for 0.61–2.27%, 0.19–0.84%, 0.92–1.20%, 2.33–6.12%, 0.14–0.50%, and 0.08–0.13% of the total bacteria OTUs for each sample.

### Co-occurrence network construction and analysis

**Figure 5:**
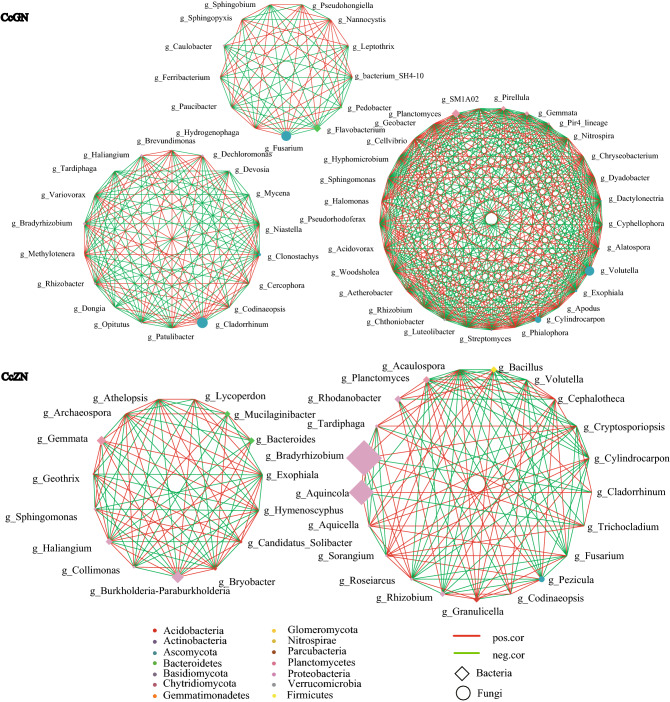
Microbial network in the endosphere of healthy and diseased Cc*.* The microbial network was constructed by Cytoscape 3.8.0. Microbial network in the endosphere of diseased Cc (CcGN), Microbial network in the endosphere of healthy Cc (CcZN). The co-occurrence network was inferred for each maternal sample type by a pairwise correlation of relative abundance for all genera. The elliptical and square node in the network indicates a fungal and bacterial genus, respectively. Node size represents the average relative abundance of one genus in each sample type. Nodes in different colour show genera belong to the phylum. Edge colour shows positive (red) and negative (blue) correlations, respectively.

The microbial network in the endosphere of the diseased Cc consisted of 60 genera (from 13 phyla) and 578 edges (Fig. 5, CcGN). The top 10 most abundant genera included *Fusarium*, *Flavobacterium*, *Pedobacter*, *Caucobacter*, *Paucibacter*, *Ferribacterium*, *Nannocystis*, *Hydrogenophaga*, *Sphingobium,* and *Sphingopyxis*. The microbial network within the endosphere of healthy Cc consisted of 36 genera (from 14 phyla) and 208 edges (Fig. 5, CcZN). Compared with healthy Cc, CcGN had a higher clustering coefficient (0.933), total nodes (60), exclusive nodes (35), total edges (578), exclusive edges (46), and a lower average path length (1.093). This result showed that there was a tighter and more complex microbial interaction network in the endosphere of diseased plants than in healthy plants. The rhizosphere microbial network of diseased Cc consisted of 40 genera (from 13 phyla) and 190 edges (Figure S6, CcGJ). The rhizosphere microbial network of healthy Cc consisted of 40 genera (from 13 phyla) and 372 edges (Figure S6, CcZJ). The rhizosphere microbial network of diseased Cc had a higher diameter (3), a higher exclusive node (18), and an average path length (1.265) but lower density (0.244), total edges (190), and exclusive edges (170) than the healthy plants. The rhizosphere microbial interaction network of the Cc rhizosphere changed obviously after the disease. The complexity and the interaction were reduced in the diseased plants compared with healthy plants.

### Microbial isolation and functional verification

**Figure 6:**
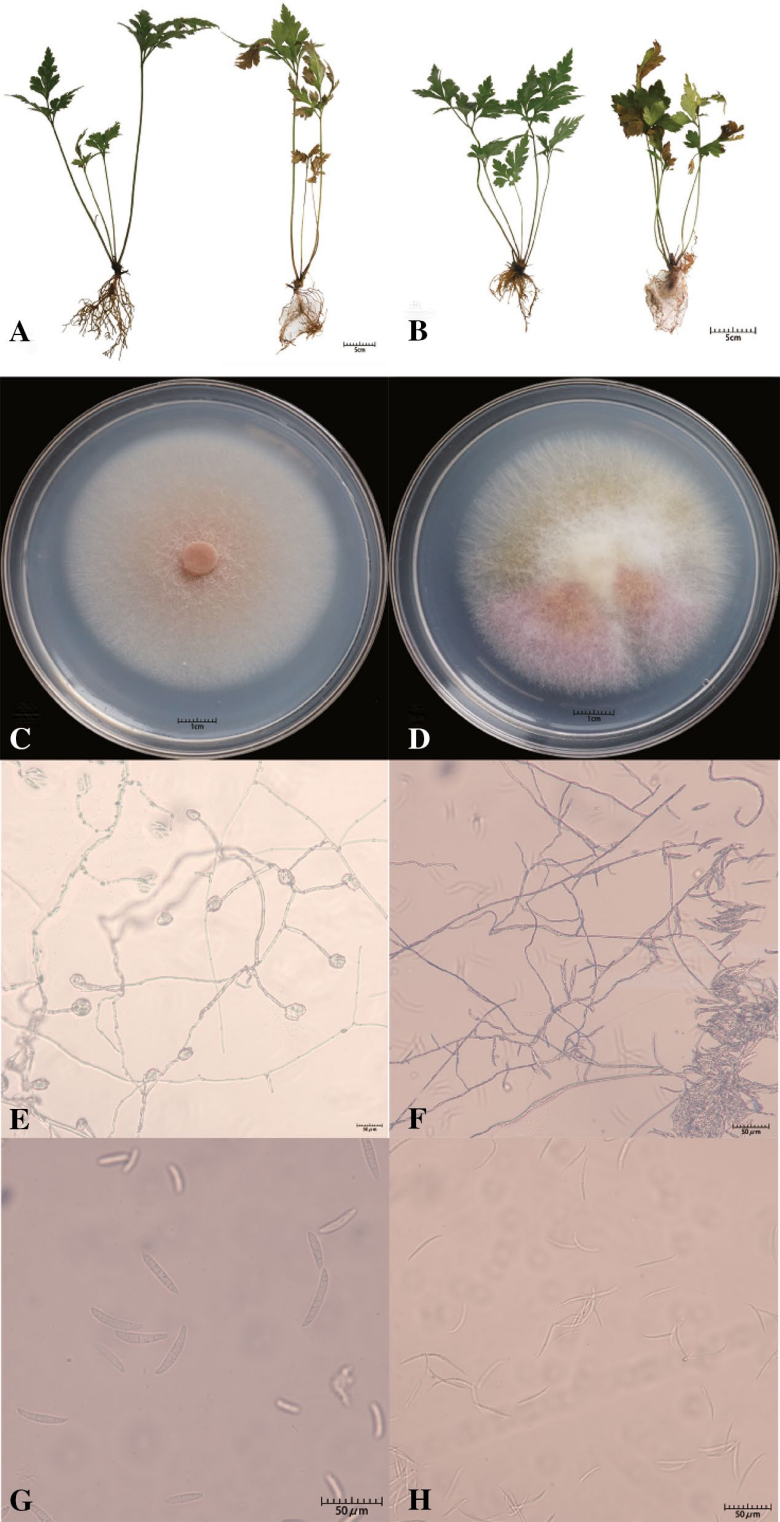
Pathogenicity determination and morphological identification of pathogenic fungi. (**A**,**B**) Pathogenicity determination of H1 and H2. The left side shows the state of the root without inoculation, and the right side shows 7 days after inoculation. (**C**,**D**) Colony characteristics on PDA of H1 and H2. (**E**,**F**) Microconidia in situ on PDA of H1 and H2. (**G**,**H**) Macroconidia in situ on PDA of H1 and H2.

Two distinct fungal isolates (H1 and H2) were isolated from the endosphere of diseased Cc (CcGN group), and Koch’s postulates were conducted to verify the pathogenicity of individual isolates (Fig. 6). The 10 repeated inoculations of two isolates resulted in root rot disease of Cc, with a 100% incidence rate. The isolates were identified using internal transcribed spacer (ITS) and translation elongation factor 1α (EF-1α) rRNA molecular analysis and morphological characteristics. H1 was identified as *F. solani*, and H2 was identified as *F. avenaceum* (Figure S7).

**Figure 7:**
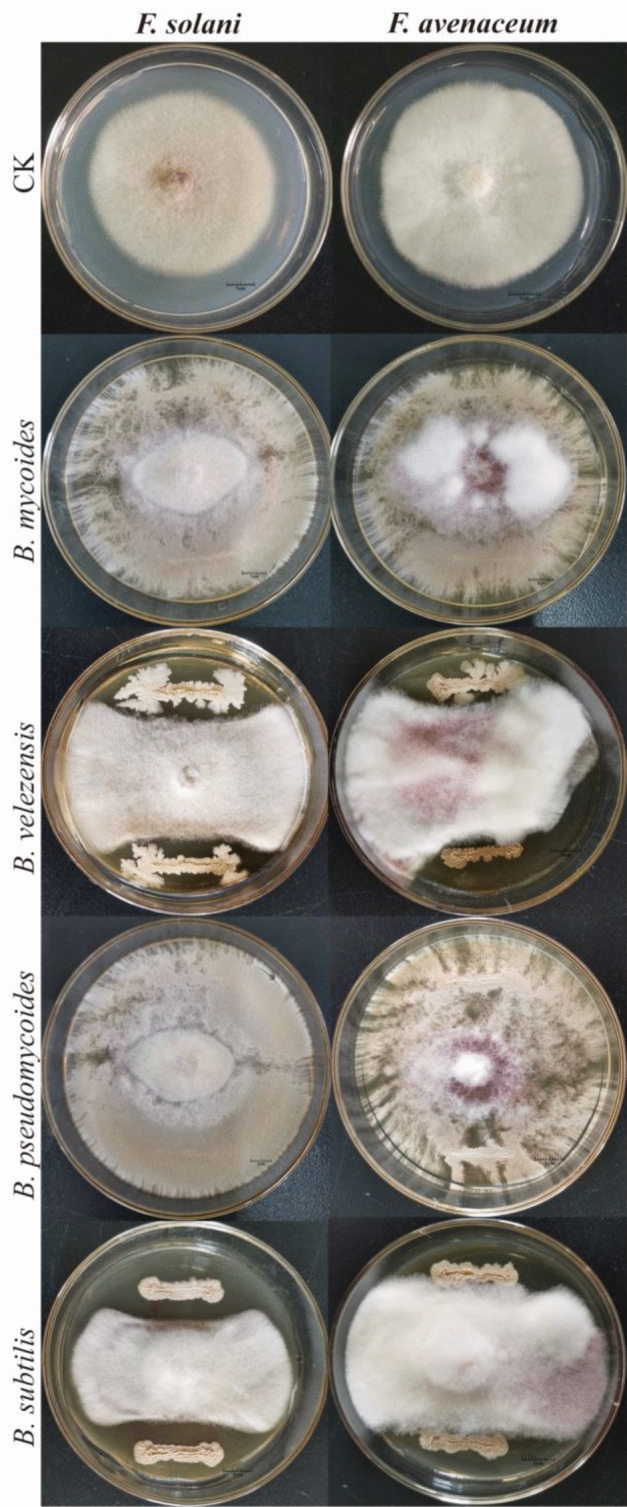
Bacteria with antagonistic activity against root rot pathogens of Cc by the dual culture method.CK: without the antagonistic bacteria.

**Figure 8:**
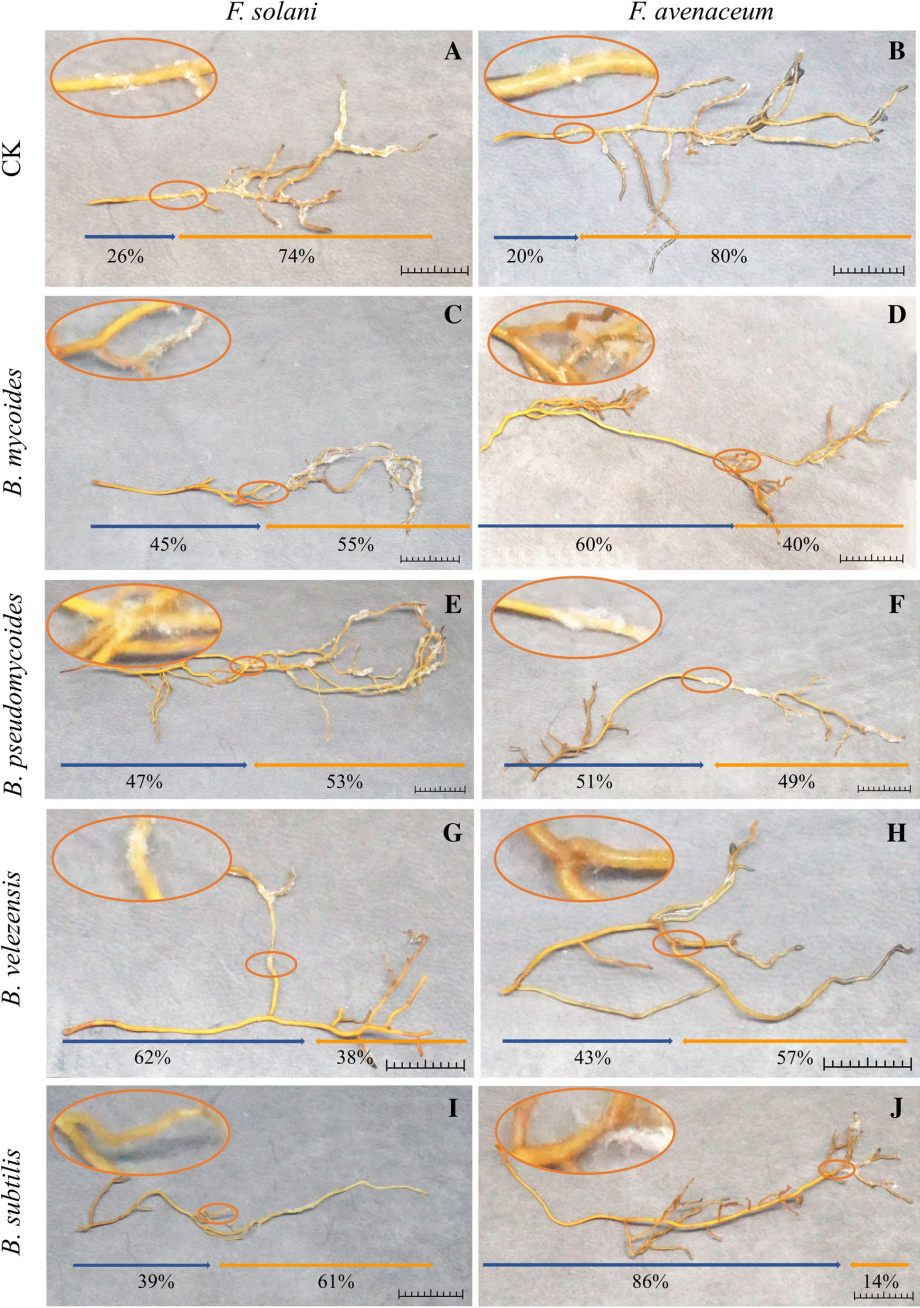
Bacteria with antagonistic activity against root rot pathogens of Cc in detached roots. The place within the leftmost (base side) 10 mm of fibrous roots were inoculated with 100 μL sterile water (CK, **A**,**B**), and suspension of *B. mycoides* (**C**,**D**), *B. pseudomycoides* (**E**,**F**), *B. velezensis* (**G**,**H**), and *B. subtilis* (**I**,**J**), The place within the rightmost (apical side) 10 mm of fibrous roots were inoculated with 100 μL spore suspension of *F. solani* (**A**,**C**,**E**,**G**,**I**) and *F. avenaceum* (**B**,**D**,**F**,**H**,**J**). The length of the orange arrow represents the proportion of root colonized by fungus. The length of the blue arrow represents the proportion of root colonized without fungus. Scale bar 1 cm.

Based on KOMODO & GROWREC recommended media, 14 *Bacillus* strains were isolated, which produced obvious inhibition bands to the two pathogens on PDA medium and detached roots (Figs. 7, 8). 14 *Bacillus* strains with antagonistic activity against root rot pathogens of Cc were isolated from the endosphere of healthy Cc. They were identified as *B. subtilis* (830-002, JM-003), *B. velezensis* (JM-001, JM-002, JM-005), *B. pseudomycoides* (LB-016, LB-050, LB-070, LB-074, NA-012, NA-048, YEM-014), and *B. mycoides* (LB-013, LB-021) based on 16S rRNA molecular identification (Figure S8). Four kinds of *Bacillus* bacteria produced obvious inhibition bands against the two pathogens on PDA medium and detached roots. This indicated that they had an obvious inhibitory effect on two root rot pathogens of Cc.

## Discussion

Previous studies on Cc root rot are mostly based on culture-dependent methods to isolate and identify a variety of pathogens of the genus *Fusarium*^29–32^. There were also two studies using high-throughput sequencing technology to study the bacterial and fungal community diversity in the rhizosphere of Cc^1, 33^. It was found that the bacterial richness and diversity of the rhizosphere significantly decreased in diseased versus healthy plants, and the significant increase in the abundance of *Fusarium* was the main reason for the occurrence of root rot. However, Koch’s postulates state that pathogens can be isolated and cultured from the host, then, the pathogenic microorganisms can reinfect the host and cause the host to have the same symptoms, and the same pathogen can be isolated from the symptom site again. Therefore, real pathogenic microorganisms must be found in the endosphere. Based on previous studies, we have increased the research on microorganisms in the endosphere. Based on the results, we preliminarily understand the microbial composition and function in the endosphere and rhizosphere and explore the possible pathogenic microorganisms of Cc root rot and the beneficial microorganisms that can be used for disease control.

The microbial diversity showed that the α-diversity of the rhizosphere fungi of the diseased plants was significantly higher than that of the healthy plants, and the α-diversity of the bacteria in the endosphere of the diseased plants was significantly higher than that of the healthy plants. The pathogens destroyed the plant tissue and entered the rhizosphere soil, which led to an increase in the α-diversity of rhizosphere fungi. The diseased tissue may recruit more bacteria to resist the infection of pathogens, which leads to an increase in bacterial α-diversity in the endosphere of diseased plants. PCoA and NMDS analysis suggested this, which showed that the root microbiome of Cc was significantly affected by disease and niche.

FUNGuild was used to predict the function of fungi in the endosphere and rhizosphere of Cc. It was found that the saprotroph type in the endosphere of diseased plants was significantly higher than that in the endosphere of healthy plants. Further analysis of the functional groups for the fungal community revealed that the animal pathogens, soil saprotrophs, plant saprotrophs, and wood saprotrophs were significantly higher than those in the endospheres of healthy plants. The plant pathogens in the rhizosphere of diseased plants were significantly higher than those of healthy plants. Therefore, these fungi may be the main pathogens of Cc root rot. PICRUSt2 predicted the function of bacteria and found that the bacteria in the endosphere of the diseased plant could obtain more nutrient resources than healthy plants, and that the metabolism of the bacteria was stronger. The bacteria in the endosphere had obvious interactions and tended to adapt to changes in the environment. These results indicated that the root system of Cc might recruit more microorganisms to fight the pathogenic bacteria of root rot. There are many beneficial microorganisms, such as *Bacillus*, in the rhizosphere of plants, which can not only promote the growth of plants but also help plants resist the invasion of pathogens. Among the plant microbial communities, the number of bacterial communities is much larger than that of the fungal community, so a small number of fungal community changes can be detected. In addition, the rhizosphere is a buffer zone in a state of equilibrium, in which the abundance of the microbial community is much higher than that in the endosphere, so a little number of microbial community changes are difficult to be detected. That is why no significant difference in the function of the rhizosphere microbial community between healthy and diseased Cc.

Indicator species analysis found that the endosphere of the diseased plants was significantly enriched in *Fusarium*, *Volutella*, *Cladorrhinum*, *Cylindrocarpon*, *Exophiala,* and *Flavobacterium*. According to the literature reports, *Fusarium* is one of the most important groups of plant pathogenic fungi, which causes a series of diseases in a variety of crops and affects the diversity of crops in various climate regions around the world^34^. *Fusarium* is also the main culprit of *Coptis* root rot reported in the literature^3–7, 35, 36^. *Volutella* are pathogens that cause Volutella blight in a variety of hosts^29, 30, 37^. *Cylindrocarpons* are important pathogens that cause rust rot in ginseng and American ginseng^38^. Most of the studies on *Exophiala* have reported them as pathogens of animals and humans^39, 40^. *Cladorrhinum* is a biological control fungi (*Cladorrhinum foecundissimum*) and can reduce viral diseases caused by *Rhizobia* and *Pythium*^31^, and the endophytic bacteria *Cladorrhinum foecundissimum* can prevent cotton root rot caused by *Rhizoctonia solani*^41^. Members of *Flavobacterium* can promote plant growth by producing auxin, gibberellin, and cytokinin^42, 43^. Their powerful combination of extracellular enzymes is believed to be related to the degradation of bacteria, fungi, insects, and nematodes in the environment. They play an important role in plant defense^44^. Therefore, the pathogenic microorganisms of Cc root rot are not only *Fusarium* fungi but also other microorganisms that cause root rot or are accomplices of *Fusarium*, such as *Volutella*, *Exophiala*, and *Cylindrocarpon* fungi.

Poudel et al.^45^ pointed out that the co-occurrence network analysis of plant microbial communities could provide a new perspective for strengthening disease management and identifying candidate microorganisms affecting plant health. The results showed that there were significant differences in the microbial symbiosis network in the endosphere and rhizosphere of healthy and diseased Cc. Compared with the microbial network in the endosphere of healthy plants (CcZN), the microbial network in the endosphere of diseased plants (CcGN) had a tighter and more complex microbial interaction network. Ecological networks are a standard method for describing and analyzing direct interactions among species. The network structure controls the stability of ecological communities^46^, including their ability to be invaded by new species^47^. Through the microbial network, it was found that *Bacillus*, *Planctomyces*, *Rhizobium*, and *Roseiarcus* were all negatively correlated with *Fusarium*, *Volutella,* and *Cylindrocarpon* in the healthy endosphere. According to the literature reports, many *Bacillus* species have been proven to be effective against a wide range of plant pathogens^48^. They have been reported as plant growth promoters and systemic resistance inducers and produce a wide range of antibacterial compounds (lipopeptides, antibiotics, and enzymes)^49^ and growth factor (space and nutrients) competitors^50^. Compounds with antifungal and antibacterial activities can be isolated from *Planctomyces* bacteria^51, 52^. *Rhizobium*, as a biological control bacterium for plant root rot^53–55^, has an antagonistic effect on fungi^56^ and promotes host growth^57^. *Roseiarcus* are mycorrhiza helper bacteria (MHB)^58^ that can spread to root tips^59^ and promote the growth and colonization of mycorrhizae^60, 61^. We believe that *Bacillus*, *Planctomyces*, *Rhizobium*, and *Roseiarcus* may be the key microorganisms to maintain the healthy growth of *Cc*.

Considering the prevalence of *Fusarium* in root rot of Cc and the high relative abundance of *Bacillus* in healthy plant roots, selective isolation and functional verification of *Fusarium* and *Bacillus* were carried out*. F. solani* and *F. avenaceum* have been isolated and verified as pathogens of Cc root rot based on Koch’s postulates. *F. solani*^3^ and *F. avenaceum*^7^ have been reported in other articles as pathogens of Cc, which also proves the correctness of our conjecture. However, we did not isolate any fungi belonging to *Volutella*, *Exophiala*, and *Cylindrocarpon*, and we cannot determine whether they are also pathogenic to *Coptis* or cause root rot because more isolation methods are needed to culture them. From the healthy endosphere, 14 *Bacillus* strains with antagonistic activity against root rot pathogens of Cc were isolated. They were identified as *B. subtilis*, *B. velezensis*, *B. pseudomycoides*, and *B. mycoides* by 16S rRNA molecular identification. The accuracy of our conjecture on antagonistic microorganisms of Cc root rot was partly proven. The strategy of high-throughput sequencing has the potential for comprehensive identification of pathogenic and antagonistic microorganisms for plant disease.

In this study, the ITS2 fragment and 16S V3–V4 fragment approximately were 250 bp and 400 bp, respectively. Maybe the short-read cannot covering the full length of the 16S or ITS rRNA gene, which often causes ambiguity in taxonomic classification, and not enough to identify the sequence on the species level^62^. Due to lack of genomic information of *C. chinensis*, amplicon sequencing is the useful method to check the microbial community in the endosphere of *C. chinensis*. In addition, no results reported in interaction of endosphere in *C. chinensis*, especially in identification of antagonistic microorganisms. Furthermore, the other methods, for example, full-length amplicon sequencing and metagenetic sequencing will be used in this field in our further research to achieve this goal. These technologies can be used for the auxiliary rapid identification of pathogenic microorganisms. It is necessary to identify the pathogen by traditional means and verify it by Koch's postulates. However, high-throughput sequencing can point out the direction for the isolation of pathogenic bacteria, especially for a microorganism that has no effective method for pure culture. More importantly, high-throughput sequencing can be used to understand the composition of the microbial community and construct a microbial interaction network. Through the microbial interaction network, we can identify and obtain microorganisms that are antagonistic to pathogens.

## Methods and materials

### Experimental design and sampling

#### Collection and processing of experimental materials

Healthy and root rot-diseased Cc roots from the same field were taken and got permission from the Cc cultivation bases of Wawushan Pharmaceutical Co., Ltd. in Hongya County (Group 2, Heishan Village, Gaomiao Town, Hongya County, Meishan City, Sichuan Province, China, 29° 29′ 10.91″ N, 103° 9′ 39.9″ E) in November 2018. Five healthy or diseased plants were mixed as a duplicate sample. The healthy and diseased samples were repeated 3 times, respectively, then samples were packed in a sterile plastic bag and put into a fresh-keeping box with ice packs before arriving in the laboratory for immediate disposal. The criteria for choosing the diseased Cc plants were as follows. First, plants with withered leaves aboveground were selected. Then, the plants were pulled out, and the plants with root rot were selected as the diseased plants. The voucher specimen of the Cc plant roots was approved by Prof. Yuntong Ma at Chengdu University of Traditional Chinese Medicine, and samples of the plant roots (voucher specimen codes 511452181103LHL0001 to 511452181103LHL0006) were deposited in the State Key Laboratory Breeding Base of Systematic Research, Development and Utilization of Chinese Medicine Resources, Chengdu University of Traditional Chinese Medicine, Chengdu, China.

The loose soil around the roots of Cc was shaken off until only soil adhered to the root surface, and the fibrous roots were cut and placed in a sterile 50 mL centrifuge tube. The rhizosphere soil on fibrous roots were released by sonication using a 30-s pulse–30-s off-cycle repeated 4 times. The suspension was centrifuged, and the supernatant was discarded from the rhizosphere soil. The fibrous root samples described above were surface sterilized by consecutive immersion for 30 s in 75% ethanol and 10 min in 2% sodium hypochlorite, followed by 4 rinses in sterile distilled water. The rhizosphere soil and the fibrous root were quickly frozen in liquid nitrogen and stored in a freezer at − 80 °C until required for DNA extraction. All methods involving plants were carried out in accordance with relevant national, international, or institutional guidelines and regulations.

#### DNA extraction and PCR amplification

Microbial DNA was extracted using HiPure Soil DNA Kits (Magen, Guangzhou, China) according to the manufacturer’s protocols. The 16S rDNA V3-V4 region of the ribosomal RNA gene was amplified by PCR using primers 341F: CCTACGGGNGGCWGCAG and 806R: GGACTACHVGGGTATCTAAT^63^. The fungal ITS2 region was amplified using the primers ITS3_KYO2: GATGAAGAACGYAGYRAA and ITS4: TCCTCCGCTTATTGATATGC^64^. PCRs were performed in triplicate in a 50 μL mixture containing 5 μL of 10 × KOD Buffer, 5 μL of 2 mM dNTPs, 3 μL of 25 mM MgSO_4_, 1.5 μL of each primer (10 μM), 1 μL of KOD Polymerase, and 100 ng of template DNA. The reaction conditions were as follows: 94 °C for 2 min, followed by 30 cycles at 98 °C for 10 s, 62 °C for 30 s, and 68 °C for 30 s and a final extension at 68 °C for 5 min.

#### Illumina Hiseq 2500 sequencing

Amplicons were extracted from 2% agarose gels and purified using the AxyPrep DNA Gel Extraction Kit (Axygen Biosciences, Union City, CA, USA) according to the manufacturer’s instructions and quantified using an ABI StepOnePlus Real-Time PCR System (Life Technologies, Foster City, USA). Purified amplicons were pooled in equimolar amounts and paired-end sequenced (2 × 250) on an Illumina platform according to standard protocols^65^. The raw reads were deposited into the NCBI Sequence Read Archive (SRA) database (Accession Number: SRP286246).

#### Isolation and characterization of the pathogens

In November 2018, 33 diseased roots from the same field with high-throughput sequencing samples were collected from Sichuan (29° 29′ 10.91″ N, 103° 9′3 9.9″ E), and small samples (0.5 cm in length) were cut from the border between diseased and healthy tissue, successively sterilized with 75% ethanol and 2% sodium hypochlorite, rinsed 3 times in sterilized water, dried on sterilized filter paper, transferred onto PDA, and incubated at 25 °C for 7 days in the dark. Two distinct fungal isolates (H1, H2) were isolated, and Koch’s postulates were conducted to verify the pathogenicity of individual isolates. The isolates were identified using internal transcribed spacer (ITS) and translation elongation factor 1α (EF-1α) rRNA molecular analysis and morphological characteristics.

#### Isolation, identification, and activity of antagonistic bacteria

Antagonistic microorganisms were isolated using the media that KOMODO & GROWREC (http://komodo.modelseed.org/default.htm) recommended based on 16S rRNA or the genus name. The antagonistic interaction between antagonistic microorganisms and pathogens was studied on PDA media by the dual culture method. PDA medium without antagonistic bacteria was used as the negative control (CK). The active isolates were identified based on their 16S rRNA molecular analysis using PAUP * 4.0 Beta 10 with the maximum parsimony method. Then, the identified bacteria were used to measure the antagonistic ability on the detached root, with sterile water used as a negative control (CK). The place within the rightmost (apical side) 10 mm of fibrous roots were inoculated with spore suspension of two kinds of pathogens (2.1 × 10^7^ CFU/mL, 100 μL), The place within the lefttmost (base side) 10 mm of fibrous roots were inoculated with a corresponding *Bacillus* strain (OD_600_ = 1, 100 μL). After 7 days of moistening culture, the incidence of detached roots were observed.

### Data analysis

#### 16S and ITS2 rRNA gene bioinformatics process

The Illumina MiSeq platform was used to identify the questionable sequence, USEARCH and VEARCH 2.14 were used to merge the double-ended sequences, and the primers and quality control were removed. The sequences were de-redundant and OTU was generated according to 97% similarity, and the effective sequence of each sample was obtained. We called USEARCH for annotating species, 16S rDNA V3-V4 region sequence based on Silva (Version 138)^66, 67^ and the ITS2 region sequence based on UNITE (Version 04.02.2020)^68^. After removing chimera sequences and annotating species, we removed chloroplasts and mitochondria, and the host ITS2 sequence and tag number did not exceed 5 sequences in total.

#### Diversity and statistical analysis

The Shannon α-diversity index was calculated in QIIME (version 1.9.1). The R project “ggplot2” package^69^ (version 2.2.1) was used to draw a histogram. The Shannon index between the two groups was analyzed by Welch's T test^32^. Muscle^70^ (version 3.8.31) was used for sequence alignment, FastTree^71^ (version 2.1) was used to construct a phylogenetic tree, and then we performed Bray–Curtis in the R project “Vegan” package^32^ (version 2.3.5). Multivariate statistical analysis of PCoA and NMDS of Bray–Curtis distance was performed and plotted in the “ggplot2” package^69^ with Welch's T test.

#### Analysis of functional differences

PICRUSt2^28^ was used to predict the functional genes of the bacterial community, and FUNGuild^27^ was used to predict fungal community function. The statistical analysis of STAMP software^72^ was used to detect the significant difference in the abundance of functional genes/pathways corresponding to the healthy group and the diseased group. Welch’s T test (P < 0.05) and Benjamini–Hochberg's false discovery rate (FDR) multiple test correction were applied to generate extended error bar graphs or draw statistical stacked shock graphs. The R project “ggalluvial” package (Version 0.12.2) was used to draw the fungal function stacked shock map.

#### Indicator species analysis

The R language “labdsv” package was used to calculate the indicator value of each group of species with abundance value > 0 and total proportion > 0.1% in each sample of the comparison group. Cross-validation for statistical tests was used to obtain the P value. The data are displayed as a bubble chart, and the biomarker of each group can be found intuitively by the size of the bubble.

#### Species network construction and key taxa analysis

The genera with relative abundances greater than 1% identified in the rhizosphere and endosphere of healthy and diseased *Coptis* plants were selected. We merged each group of fungi and bacteria and used the CoNet plug-in^73^ of Cytoscape (v3.8.0) software^74, 75^ for network analysis. “Pearson correlation” and “Spearman correlation” were specified as 0.7 to construct a microbial interaction network. A permutation test was performed to obtain the P value and filter out invalid edges with a P value greater than 0.01. To calculate the statistical significance of co-occurrence and mutual exclusion, we calculated the edge-specific permutation and bootstrap score distribution for 1000 iterations to obtain the final network^76^ and visualized the network through Cytoscape (v3.8.0).

## Supplementary Information

Supplementary Information 1.
