# Supplementary material for: Auxiliary rapid identification of pathogenic and antagonistic microorganisms associated with Coptis chinensis root rot by high-throughput sequencing
Source: Sci Rep. 2021 May 27;11:11141. doi: 10.1038/s41598-021-90489-9 (PMC8160328; doi:10.1038/s41598-021-90489-9)
Supplement: Supplementary file 1 — Supplementary Information 1. [file 41598_2021_90489_MOESM1_ESM.docx]

Auxiliary rapid identification of pathogenic and antagonistic microorganisms associated with *Coptis chinensis* root rot by high-throughput sequencing

**Hailang Liao ^1,2^, Ling Huang ^1,2^, Na Li ^1,2^, Wenjia Ke ^1,2^, Yiqing Xiang ^1,2^ and Yuntong Ma ^1,2,3,^ ***

^1^ State Key Laboratory Breeding Base of Systematic Research, Development and Utilization of Chinese Medicine Resources, Chengdu University of Traditional Chinese Medicine, Chengdu 611137, China

^2^ Department of Pharmacy, Chengdu University of Traditional Chinese Medicine, Chengdu 611137, China

^3^ The Engineering and Technology Research Center for the Protection and Development of Yalian Resourcesin Sichuan Province, Meishan 620360, Sichuan, China

***** Correspondence: mayuntong@cdutcm.edu.cn; Tel.: +86-139-8059-8196

**Supplementary Information**

**Supplementary Information Fig. S1-S8**

**Supplementary Information Table S1-S6**


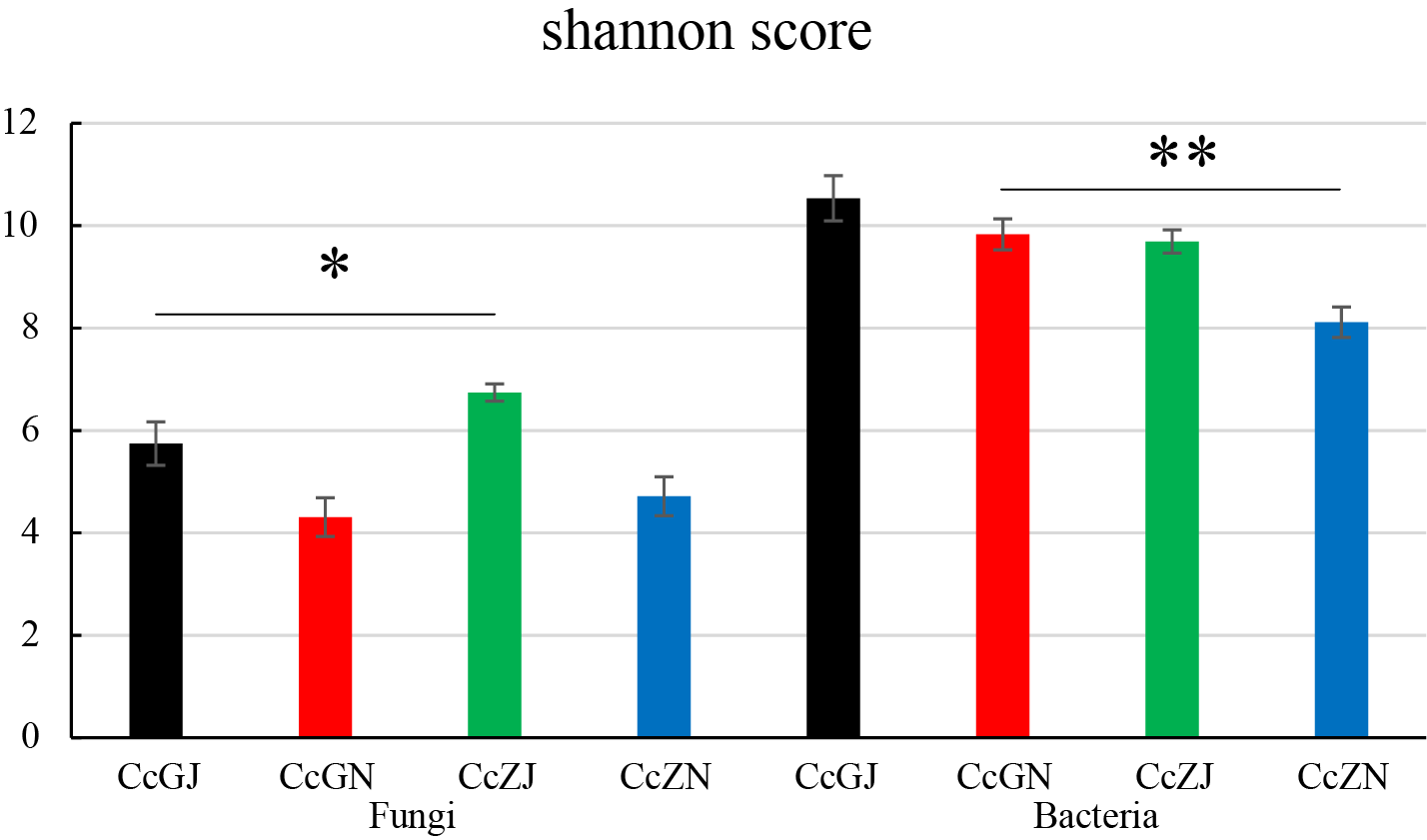


Figure S1 α Diversity in the rhizosphere and the endospheree of healthy and Diseased Cc*.* *: 0.01＜*p*≤0.05; **: *p*≤0.01.


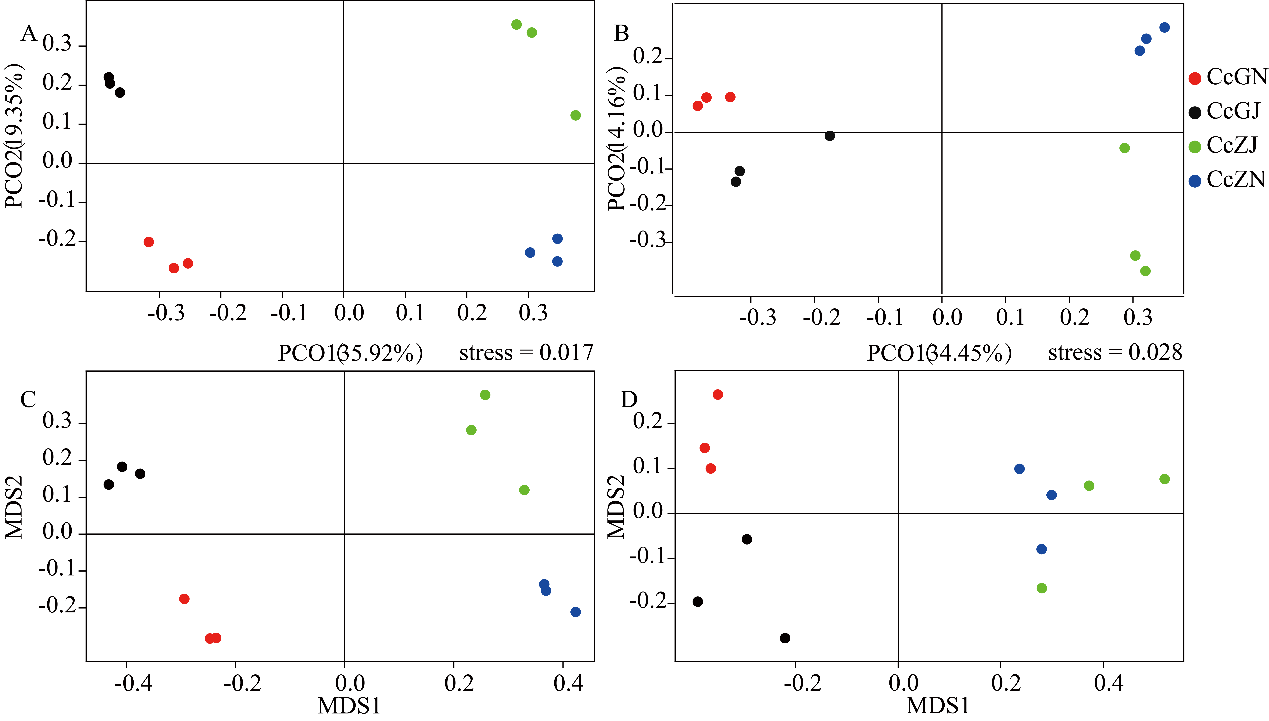


Figure S2 Analysis of the microbiome sample distance in the rhizosphere and the endospheree of healthy and diseased Cc*.* A: Principal Coordinate Analysis (PCoA) based on the Bray Curtis distance of bacteria between samples; B: Principal Coordinate Analysis (PCoA) based on the fungal Bray Curtis distance between samples. The root microbiome of Cc. was separated on the first axis of PcoA (PCo1) according to whether it was diseased or not, and the second axis (PCo2) was separated according to niche, with significant differences between groups (PERMANOVA, P<0.001); C: Non-metric multidimensional scaling analysis (NMDS) based on the Bray Curtis distance of bacteria between samples. The root microbiome of Cc. is separated on the first axis of PcoA (MDS1) according to whether it is diseased or not, and the second axis is separated according to niche, stress=0.017<0.1; D: Non-metric multidimensional scaling analysis (NMDS) based on the Bray Curtis distance of bacteria between samples. The root microbiome of Cc is separated on the first axis of NMDS (MDS1) according to whether it is diseased or not, stress=0.028<0.1.


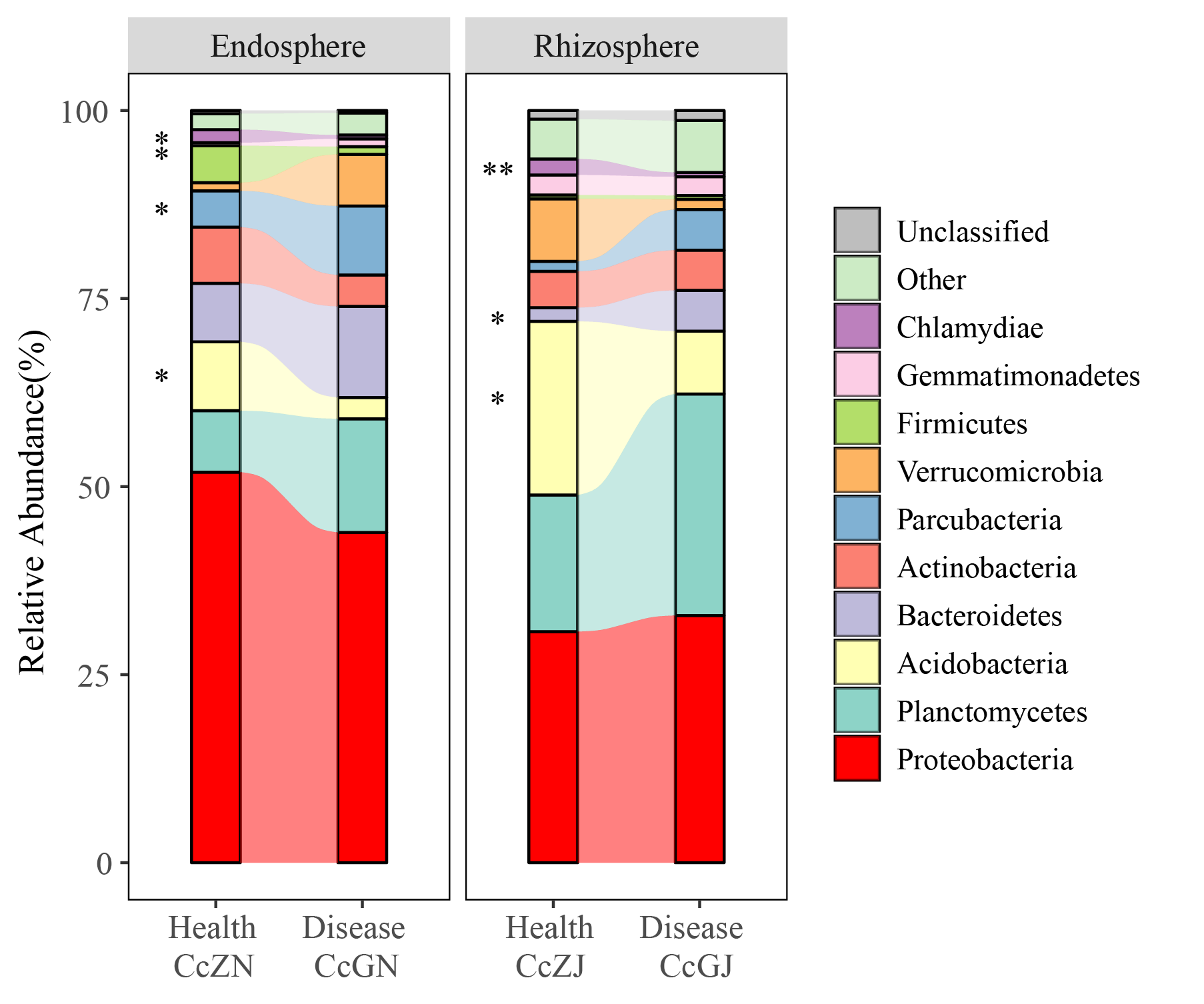


Figure S3 Stacked shock map of bacteria distribution in the rhizosphere and the endospheree of health and diseased Cc*.* *:0.01<P≤0.05; **: P≤0.01（Welch\'s t test）.


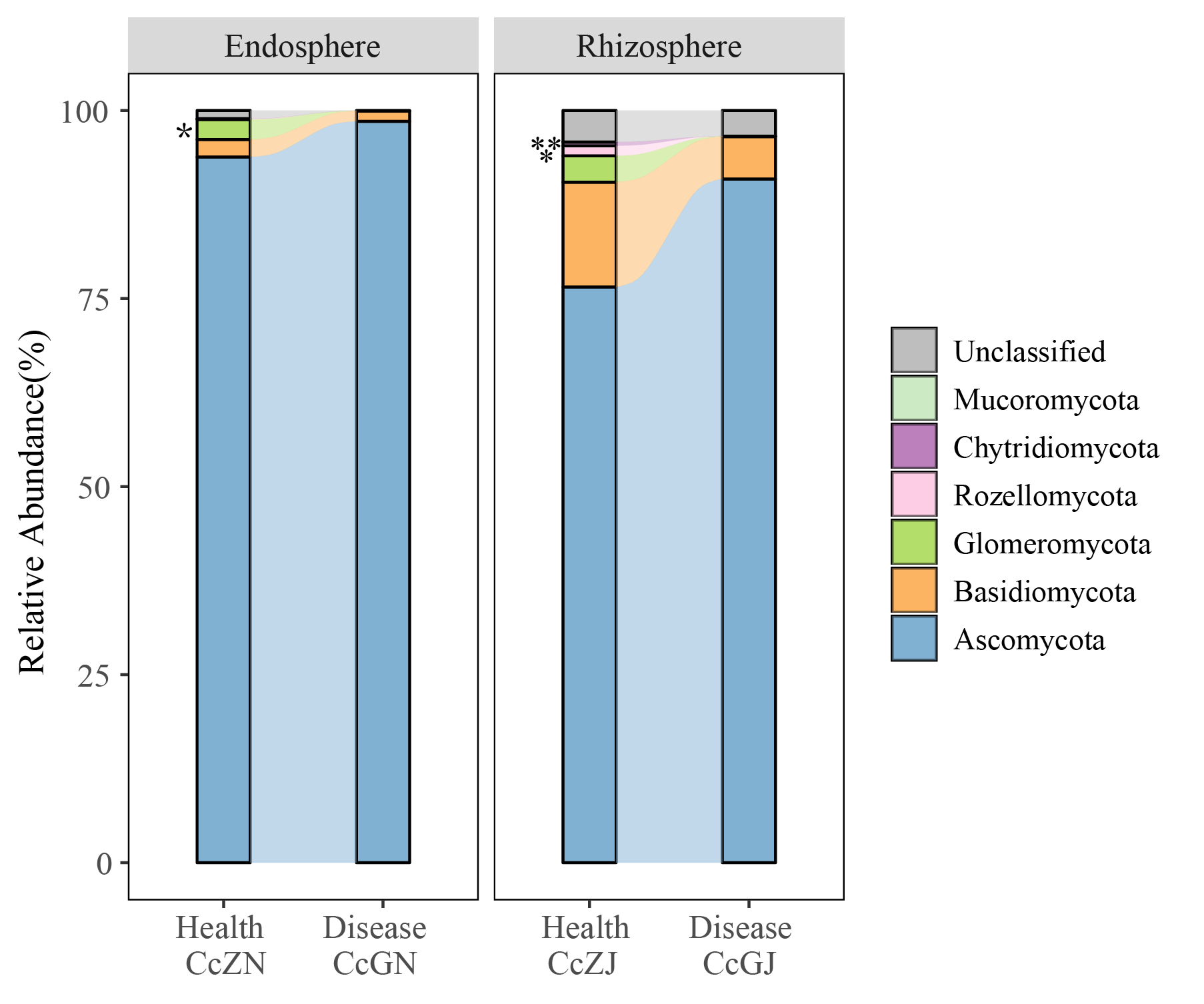


Figure S4 Stacked shock map of fungi in the rhizosphere and the endospheree of healthy and diseased Cc. *:0.01<P≤0.05; **: P≤0.01（Welch\'s t test）


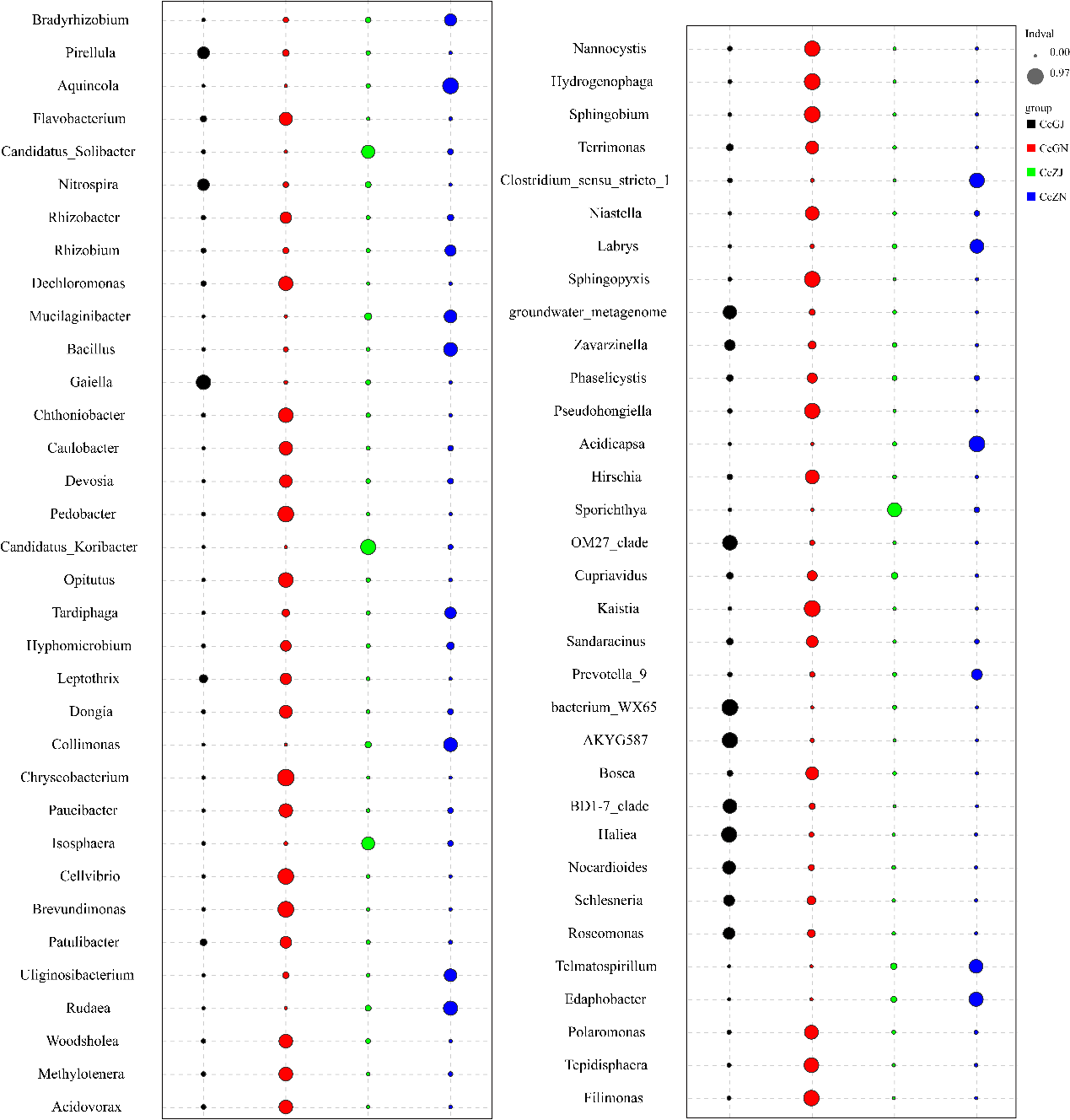


Figure S5 Analysis of indicator species of bacteria in the rhizosphere and the endosphere of health and diseased Cc. Node size represents the average relative abundance of one genus in each sample type.


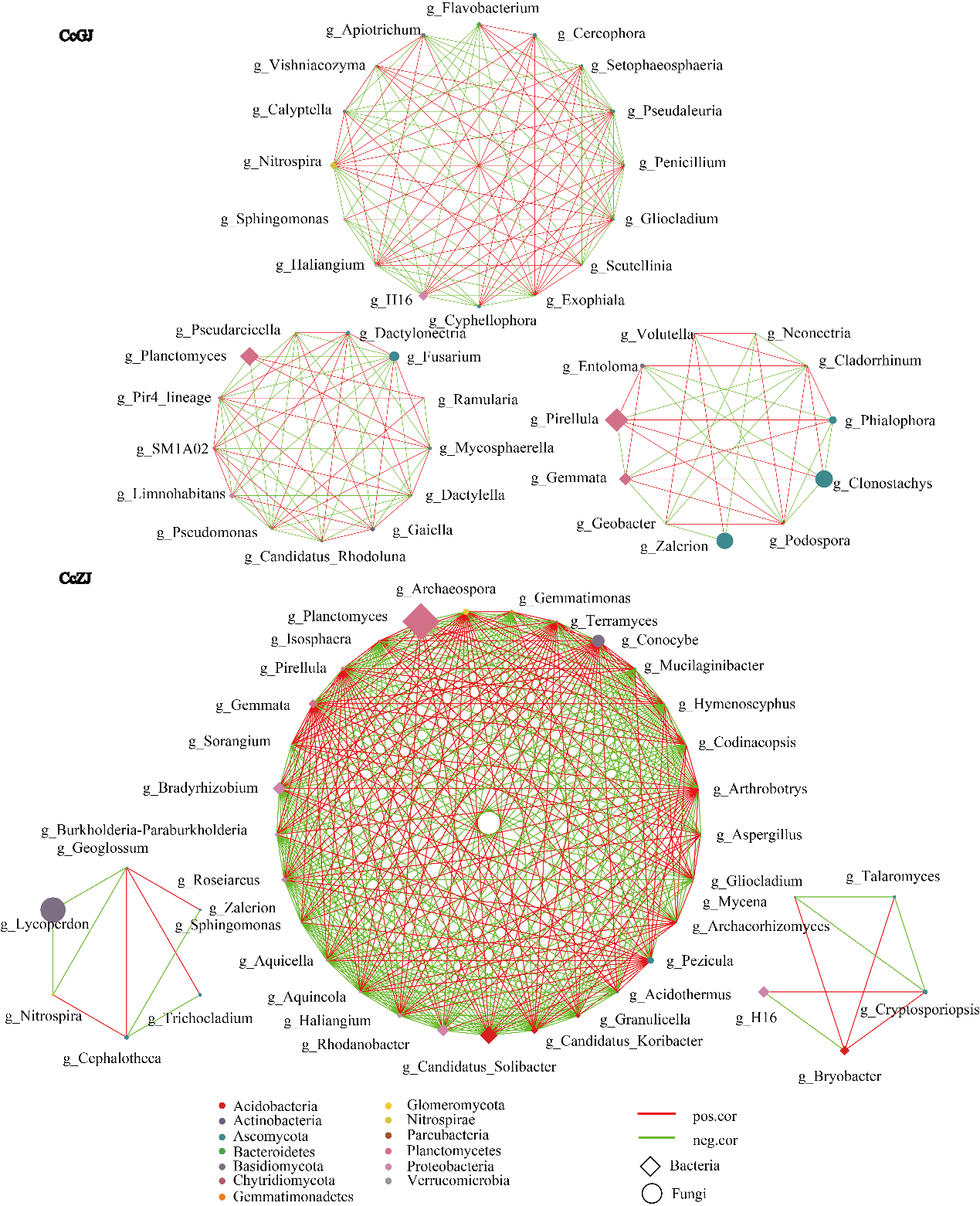


Figure S6 Microbial network in the rhizosphere of health and diseased Cc*.* The microbial network was constructed by Cytoscape 3.8.0. Microbial network in the rhizosphere of diseased Cc (CcGJ), Microbial network in the rhizosphere of healthy Cc (CcZJ). The co-occurrence network was inferred for each maternal sample type by a pairwise correlation of relative abundance for all genera. The elliptical and square node in the network indicates a fungal and bacterial genus, respectively. Node size represents the average relative abundance of one genus in each sample type. Nodes in different colour show genera belong to the phylum. Edge colour shows positive (red) and negative (blue) correlations, respectively.


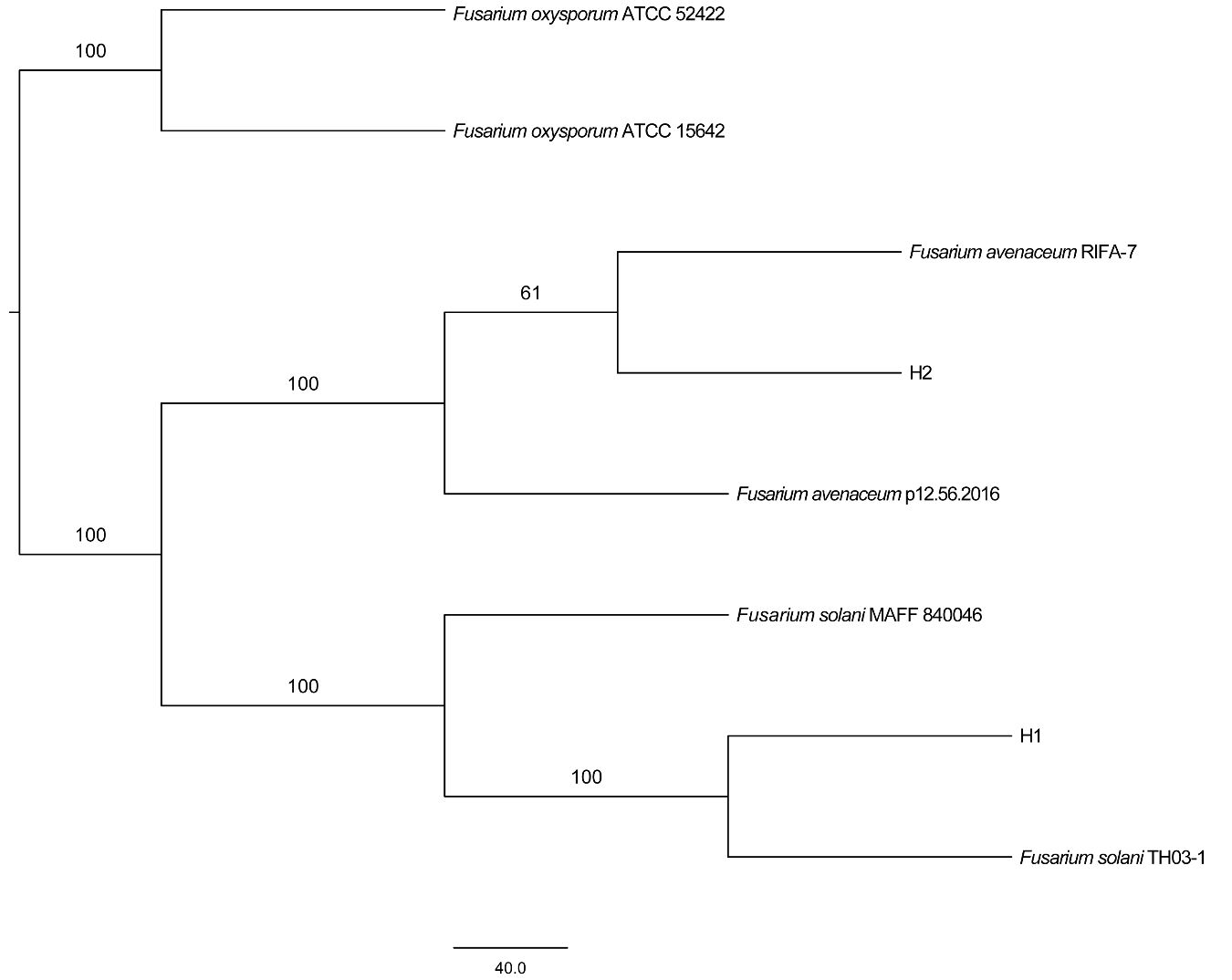


Figure S7 Phylogenetic tree based on ITS and EF1-α RNA sequences


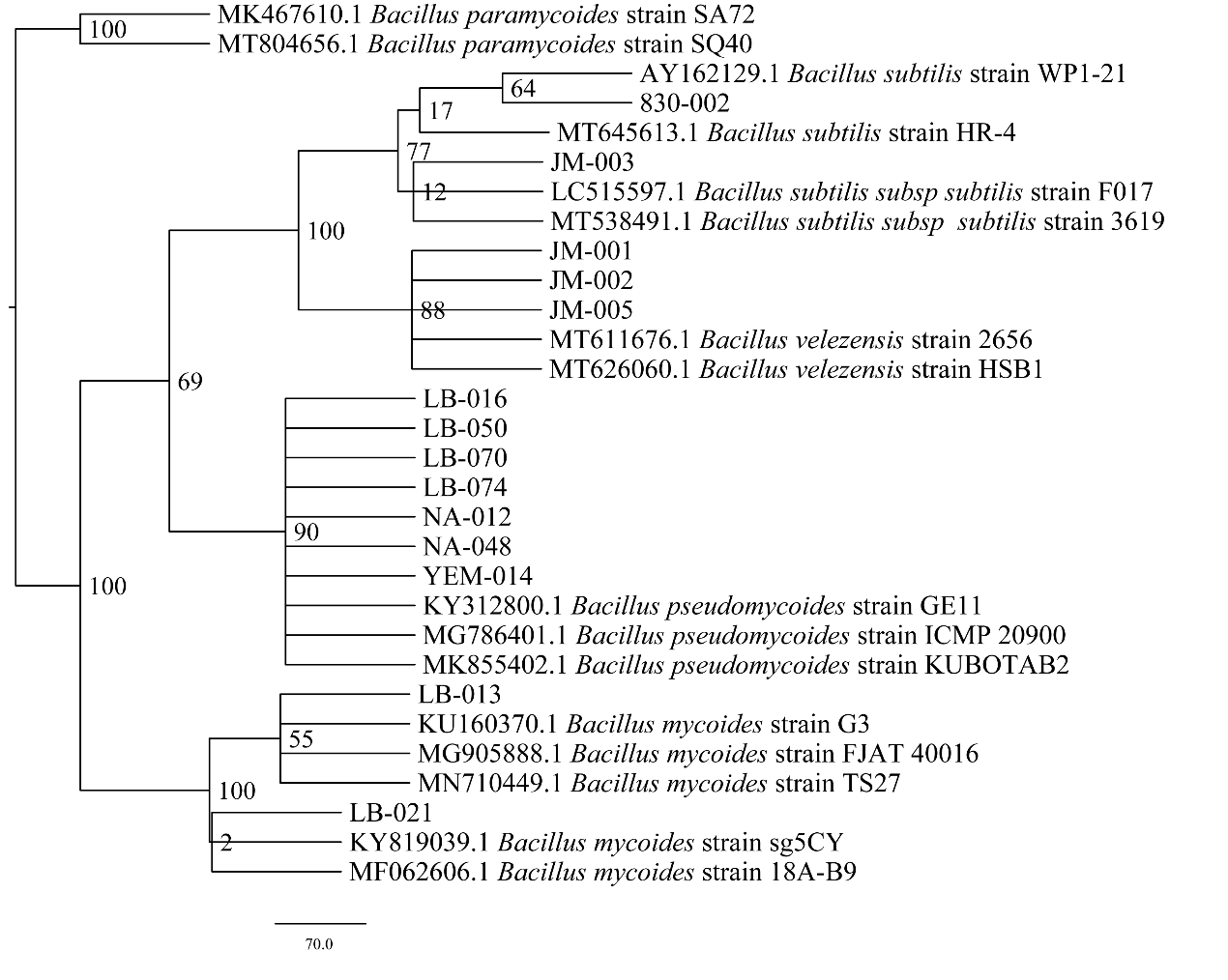


Figure S8 Phylogenetic tree based on 16s RNA sequences

| Table S1 Bacterial data pre-processing statistics and quality control | | | | | | |
| --- | --- | --- | --- | --- | --- | --- |
| Sample Name | Raw PE | Clean PE | Raw Tags | Clean Tags | Effective Tags | Effective Ratio (%) |
| CcZJ-1 | 147051 | 143301 | 143164 | 142298 | 135640 | 92.24 |
| CcZJ-2 | 124267 | 121075 | 120918 | 120123 | 119265 | 95.97 |
| CcZJ-3 | 119689 | 116160 | 116041 | 115198 | 114837 | 95.95 |
| CcGJ-1 | 109032 | 106155 | 105881 | 105033 | 104366 | 95.72 |
| CcGJ-2 | 113682 | 110485 | 110273 | 109481 | 107116 | 94.22 |
| CcGJ-3 | 117284 | 114211 | 113734 | 112890 | 112191 | 95.66 |
| CcZN-1 | 109786 | 106819 | 106721 | 106110 | 105642 | 96.23 |
| CcZN-2 | 132217 | 129044 | 128915 | 128338 | 127527 | 96.45 |
| CcZN-3 | 116135 | 112773 | 112677 | 112116 | 111659 | 96.15 |
| CcGN-1 | 112608 | 109339 | 109219 | 108507 | 107439 | 95.41 |
| CcGN-2 | 123894 | 120379 | 120284 | 119413 | 117387 | 94.75 |
| CcGN-3 | 111588 | 108350 | 108253 | 107486 | 105944 | 94.94 |

| Table S2 Bacteria tags detailed information table | | | | | | |
| --- | --- | --- | --- | --- | --- | --- |
| SampleID | Tags Number | Total length | Max length | Min length | N50 | N90 |
| CcZJ-1 | 135640 | 61368839 | 476 | 331 | 453 | 441 |
| CcZJ-2 | 119265 | 54156109 | 476 | 352 | 455 | 441 |
| CcZJ-3 | 114837 | 52421083 | 476 | 337 | 463 | 441 |
| CcGJ-1 | 104366 | 47806361 | 476 | 342 | 463 | 441 |
| CcGJ-2 | 107116 | 48918474 | 476 | 353 | 462 | 442 |
| CcGJ-3 | 112191 | 51376553 | 476 | 354 | 463 | 441 |
| CcZN-1 | 105642 | 47581022 | 470 | 434 | 443 | 441 |
| CcZN-2 | 127527 | 56948936 | 472 | 332 | 443 | 441 |
| CcZN-3 | 111659 | 50020192 | 474 | 302 | 443 | 441 |
| CcGN-1 | 107439 | 48748262 | 474 | 315 | 459 | 441 |
| CcGN-2 | 117387 | 53619024 | 476 | 434 | 461 | 441 |
| CcGN-3 | 105944 | 48210867 | 476 | 332 | 461 | 441 |

| Table S3 Statistics table of the number of OTUs and tags of different bacterial samples | | | | | | |
| --- | --- | --- | --- | --- | --- | --- |
| **SampleID** | **Total Tags** | **Unique Tags** | **Taxon Tags** | **Unclassified Tags** | **Singleton Tags** | **OTUs** |
| CcZJ-1 | 135640 | 108361 | 118011 | 0 | 17629 | 5766 |
| CcZJ-2 | 119265 | 98336 | 109246 | 0 | 10019 | 5480 |
| CcZJ-3 | 114837 | 91253 | 105165 | 0 | 9672 | 4538 |
| CcGJ-1 | 104366 | 89453 | 96629 | 0 | 7737 | 5744 |
| CcGJ-2 | 107116 | 81200 | 100439 | 0 | 6677 | 4608 |
| CcGJ-3 | 112191 | 92808 | 104342 | 0 | 7849 | 5457 |
| CcZN-1 | 105642 | 52224 | 102563 | 0 | 3079 | 2047 |
| CcZN-2 | 127527 | 47860 | 125660 | 0 | 1867 | 1442 |
| CcZN-3 | 111659 | 47250 | 109298 | 0 | 2361 | 1833 |
| CcGN-1 | 107439 | 69708 | 101890 | 0 | 5549 | 3637 |
| CcGN-2 | 117387 | 82155 | 110068 | 0 | 7319 | 4177 |
| CcGN-3 | 105944 | 76807 | 98632 | 0 | 7312 | 4347 |

| Table S4 Fungal data preprocessing statistics and quality control | | | | | | |
| --- | --- | --- | --- | --- | --- | --- |
| **Sample Name** | **Raw PE** | **Clean PE** | **Raw Tags** | **Clean Tags** | **Effective Tags** | **Effective Ratio (%)** |
| CcZJ-1 | 170375 | 168700 | 168191 | 160078 | 160078 | 93.96 |
| CcZJ-2 | 167581 | 165659 | 165023 | 144136 | 144136 | 86.01 |
| CcZJ-3 | 143746 | 142000 | 140131 | 126468 | 126468 | 87.98 |
| CcGJ-1 | 189725 | 187732 | 186870 | 176516 | 176516 | 93.04 |
| CcGJ-2 | 189590 | 187225 | 183677 | 168323 | 168323 | 88.78 |
| CcGJ-3 | 160955 | 159292 | 158521 | 151073 | 151073 | 93.86 |
| CcZN-1 | 190589 | 189160 | 188973 | 188760 | 188760 | 99.04 |
| CcZN-2 | 177353 | 175882 | 175733 | 175572 | 175572 | 99.00 |
| CcZN-3 | 162109 | 160642 | 160493 | 160088 | 160088 | 98.75 |
| CcGN-1 | 188370 | 186815 | 186653 | 186159 | 186159 | 98.83 |
| CcGN-2 | 236245 | 233984 | 233523 | 232232 | 232232 | 98.30 |
| CcGN-3 | 174149 | 172624 | 172469 | 172072 | 172072 | 98.81 |

| Table S5 Fungus tags details | | | | | | |
| --- | --- | --- | --- | --- | --- | --- |
| **SampleID** | **Tags Number** | **Total length** | **Max length** | **Min length** | **N50** | **N90** |
| CcZJ-1 | 160078 | 59083526 | 399 | 226 | 385 | 327 |
| CcZJ-2 | 144136 | 51976503 | 399 | 223 | 374 | 328 |
| CcZJ-3 | 126468 | 44961272 | 399 | 204 | 359 | 326 |
| CcGJ-1 | 176516 | 62071085 | 399 | 204 | 340 | 327 |
| CcGJ-2 | 168323 | 59926179 | 399 | 233 | 351 | 329 |
| CcGJ-3 | 151073 | 54536271 | 399 | 237 | 377 | 327 |
| CcZN-1 | 188760 | 72183831 | 399 | 271 | 385 | 382 |
| CcZN-2 | 175572 | 67284639 | 399 | 271 | 385 | 382 |
| CcZN-3 | 160088 | 61027759 | 399 | 271 | 385 | 382 |
| CcGN-1 | 186159 | 69879071 | 399 | 274 | 385 | 338 |
| CcGN-2 | 232232 | 81186225 | 398 | 268 | 338 | 333 |
| CcGN-3 | 172072 | 64450722 | 397 | 263 | 385 | 338 |

| Table S6 Statistics of OTUs and tags of different fungal samples | | | | | | |
| --- | --- | --- | --- | --- | --- | --- |
| **SampleID** | **Total Tags** | **Unique Tags** | **Taxon Tags** | **Unclassified Tags** | **Singleton Tags** | **OTUs** |
| CcZJ-1 | 160078 | 39280 | 158240 | 0 | 1838 | 1037 |
| CcZJ-2 | 144136 | 44328 | 142356 | 0 | 1780 | 961 |
| CcZJ-3 | 126468 | 37820 | 125239 | 0 | 1229 | 720 |
| CcGJ-1 | 176516 | 48235 | 174646 | 0 | 1870 | 886 |
| CcGJ-2 | 168323 | 45221 | 166644 | 0 | 1679 | 751 |
| CcGJ-3 | 151073 | 39277 | 149791 | 0 | 1282 | 730 |
| CcZN-1 | 188760 | 33266 | 187663 | 0 | 1097 | 410 |
| CcZN-2 | 175572 | 34919 | 174343 | 0 | 1229 | 455 |
| CcZN-3 | 160088 | 32742 | 158625 | 0 | 1463 | 441 |
| CcGN-1 | 186159 | 39215 | 184063 | 0 | 2096 | 399 |
| CcGN-2 | 232232 | 59824 | 223363 | 0 | 8869 | 459 |
| CcGN-3 | 172072 | 39076 | 169682 | 0 | 2390 | 381 |
